# Measurement-Invariant Fluid Anti-Flynn Effects in Population—Representative German Student Samples (2012–2022)

**DOI:** 10.3390/jintelligence12010009

**Published:** 2024-01-17

**Authors:** Sandra Oberleiter, Sabine Patzl, Jonathan Fries, Jennifer Diedrich, Martin Voracek, Jakob Pietschnig

**Affiliations:** 1 Department of Development and Educational Psychology, Faculty of Psychology, University of Vienna, 1010 Vienna, Austriajakob.pietschnig@univie.ac.at (J.P.); 2 International Student Assessment (ZIB), TUM School of Social Sciences and Technology, Technical University of Munich, 80333 Munich, Germany; 3 Department of Cognition, Emotion, and Methods in Psychology, Faculty of Psychology, University of Vienna, 1010 Vienna, Austria; martin.voracek@univie.ac.at

**Keywords:** Flynn effect, figural reasoning, measurement invariance, psychometric g, Cattell–Horn–Carroll (CHC) intelligence model, cognitive abilities, multi-group confirmatory factor analysis (MGCFA), secondary school students

## Abstract

Generational IQ test scores in the general population were observed to increase over time (i.e., the Flynn effect) across most of the 1900s. However, according to more recent reports, Flynn effect patterns have seemingly become less consistent. So far, most available evidence on this phenomenon has been categorized by drawing on the classic fluid vs. crystallized intelligence taxonomy. However, recent evidence suggests that subdomain-specific trajectories of IQ change may well be more complex. Here, we present evidence for cross-temporal changes in measurement-invariant figural reasoning tasks in three large-scale, population-representative samples of German secondary school students (total *N* = 19,474). Analyses revealed a consistent pattern of significant and meaningful declines in performance from 2012 to 2022. Results indicate a decrease in figural reasoning of 4.68 to 5.17 IQ points per decade (corresponding to small-to-medium effects, Cohen *d*s from 0.34 to 0.38). These findings may be interpreted as tentative evidence for a decreasing strength of the positive manifold of intelligence as a potential cause of the increasing number of recent reports about inconsistent IQ change trajectories.

## 1. Introduction

In 1968, generational IQ test score changes were, for the first time, interpreted as genuine cohort effects (Schaie and Strother 1968). These changes were subsequently systematically documented by James Flynn, whose name has since become eponymous for cognitive performance increases in the general population (Flynn 1984). Generational changes were observed to be positive over most of the 20th century, with an estimated IQ increase of three points per decade, and to be differentiated according to intelligence domains, with larger fluid than crystallized IQ gains. On a global level, these increases were observed to amount to about 30, 35, and 25 IQ points from 1909 to 2013 regarding full-scale, fluid, and crystallized intelligence, respectively (Pietschnig and Voracek 2015). Notably, these global changes appeared to be nonlinear, with some periods of strong gains being interspersed with some less substantial ones, but with all changes on balance remaining positive (i.e., all in all indicating IQ gains rather than losses).

These patterns have been broadly consistent across most of the 1900s, although the strength of gains appears to be differentiated according to countries. However, more recent observations have shown that Flynn effect patterns have seemingly become less consistent, showing a deceleration (e.g., USA: Rindermann and Thompson 2013), a stagnation (e.g., Australia: Cotton et al. 2005), or even a reversal (e.g., Denmark: Dutton et al. 2016) of the Flynn effect across different countries.

It has been hypothesized that these unexpected patterns may result from the more fine-grained assessment of cognitive abilities in modern psychometric tests, which provides a more detailed account of domain-specific ability change. Specifically, most of the available evidence about the Flynn effect has primarily been contextualized within the classic taxonomy of Cattell’s differentiation between fluid and crystallized IQ (Pietschnig et al. 2023). However, according to the presently most widely accepted conceptualization of human intelligence (namely, the Cattell–Horn–Carroll model [CHC]; Schneider and McGrew 2018), fluid and crystallized intelligence are understood as broad abilities that exist on the same level of abstraction as eight further cognitive domains, all of which are superordinate to several lower-order subordinate abilities.

Within the framework of the CHC model, Flynn effects for specific cognitive abilities have recently been shown to be differentiated in terms of stratum II and, arguably, stratum I CHC abilities (Lazaridis et al. 2022). Intriguingly, stratum II domains either showed (i) positive Flynn effects (e.g., comprehension knowledge, learning efficiency), (ii) negative Flynn effects (e.g., spatial orientation, working memory capacity), (iii) ambiguous trends (fluid reasoning, reaction and decision speed, quantitative knowledge, and visual processing), or (iv) no change (processing speed, reading and writing).

This evidence does not necessarily suggest that subdomain differentiation represents a recent phenomenon, but that it instead might be due to the increasing use of more refined intelligence tests beyond the mere assessment of fluid vs. crystallized IQ and psychometric *g* in more recent decades. Despite a predominant rise in IQ test and subtest scores over time, the available evidence suggests a negative association of the Flynn effect with psychometric g (Must et al. 2003; Woodley et al. 2014; Pietschnig and Voracek 2015; for contrasting findings, see Colom and Flores-Mendoza 2001). A first direct assessment of the association of ***g*** with test score changes supports this idea, showing tentative evidence for cross-temporal decreases in achievement *g*, which may be a necessary consequence of differing population IQ (sub-)domain trajectories (Pietschnig et al. 2023).

In the traditional approach by Cattell, prior related research has demonstrated that fluid IQ typically showed more significant and more robust gains over time than crystallized IQ (Flynn 1984; Pietschnig and Voracek 2015).

However, recent findings of domain-specific changes according to the CHC model indicate ambiguous Flynn effects for fluid/reasoning-related subdomains. For example, while some results regarding matrices tests suggest only trivial effects (*d* range = −0.002 to −0.05; Lazaridis et al. 2022) or the stagnation of IQ gains (Colom et al. 2023), others indicate a reversal of the moderate Flynn effect over time (1996–2001: *d* = 0.23; 2001–2008: *d* = −0.11; Pietschnig et al. 2021). Thus, it may be reasonable to assume that fluid intelligence change trajectories are rooted in a more fine-grained assessment of specific subdomains.

A significant challenge when assessing the meaningfulness of the Flynn effect revolves around determining whether changes in test scores reflect actual changes in the population’s ability or merely represent manifestations of differential item functioning across different assessment years (DIF). DIF refers to the phenomenon where the discrepancy in average performance between samples results from variations in the difficulty of items or their ability to differentiate between levels of ability rather than differences in actual abilities as societal norms and cultural understandings evolve. This leads individuals to approach these tests with different levels of knowledge, consequently affecting the perceived difficulty of specific items (Gonthier and Gregoire 2022). Therefore, test score changes can only be meaningfully interpreted as population ability changes rather than a measurement artifact when cross-temporal measurement invariance is established (i.e., meaning that there is no DIF, and item properties have not changed over time; (Lazaridis et al. 2022)). In the light of recent evidence for unexpected, ambiguous Flynn effect patterns, such as domain-specific and/or country-specific patterns of stagnation or reversal, some researchers have argued that the Flynn effect may genuinely change its direction overall (e.g., Dutton et al. 2016). However, whether these patterns would not be better explained by item drift or domain specificity is still being determined.

To contribute to the examination of the Flynn effects in fluid intelligence, we utilized the figural-reasoning subtest of a widely used Germanophone intelligence test battery (Berliner Test zur Erfassung fluider und kristalliner Intelligenz; BEFKI; Wilhelm et al. 2014). The data were collected in 2012, 2018, and 2022 as population-representative samples, totaling about 20,000 German secondary school students.

## 2. Materials and Methods

Before accessing any data, we preregistered the study design, the analysis plan, and the specific main study hypotheses on the Open Science Framework (OSF; https://osf.io/nd7qr, accessed on 27 December 2023). The analysis code is available at https://osf.io/f96mj/files/osfstorage, accessed on 27 December 2023.

In all, data from 19,474 secondary school students from Germany were available. Sociodemographic sample characteristics are provided in Table 1.

### 2.1. Berliner Test zur Erfassung Fluider und Kristalliner Intelligenz (BEFKI)

For this study, we examined data from the Berlin Test for the Assessment of Fluid and Crystallized Intelligence (BEFKI; Wilhelm et al. 2014), a theoretically grounded intelligence test for secondary school students. It allows for the examination of students in grades 8 through 10, irrespective of the school type they are enrolled in.

The BEFKI has been developed based on the CHC model and comprises two subscales to assess crystallized and fluid intelligence. The fluid intelligence scale consists of three subscales assessing verbal, numerical, and figural task performance. We used data from a parallel form of the figural reasoning subscale for the present study. The psychometric properties of this subscale have been shown to be satisfactory, yielding reliabilities of 0.87 (McDonald’s ω) and concurrent validities of >0.90 with fluid intelligence estimates from the cognitive ability test, a well-established German intelligence test (Heller and Perleth 2000), and associations with listening, orthography, reading, and writing test scores ranging from *r* = 0.65 to 0.69 (Wilhelm et al. 2014).

### 2.2. Procedure

Within the formal assessments of the Programme for International Student Assessment (PISA), data from three population-representative cohorts of 15-year-olds were collected in Germany in 2012 (in paper–pencil format), 2018, and 2022 (computer-based administration in these subsequent cohorts).

The 16-item figural reasoning subtest had to be completed in 14 min. Across this item set, respondents were required to recognize and apply the logical rules necessary to identify two missing geometric elements required to complete a sequence of three given geometric figures. Respondents had to select the correct elements out of three potential response alternatives for the respective missing elements. Items were scored as correct when both elements were identified correctly.

### 2.3. Statistical Analysis

Two approaches were pursued to investigate (measurement-invariant) changes in figural reasoning performance. First, we calculated all pairwise standardized mean differences (Cohen *d*) between the raw scores of the 2012, 2018, and 2022 cohorts. Second, we utilized measurement invariance analyses and latent means-based calculations derived from these to quantify IQ test score changes. This latter approach allowed us to disentangle genuine cognitive ability changes from those merely caused by item drift (e.g., due to changes in item difficulty or test administration format; see (Lazaridis et al. 2022)). Consequently, we conducted multi-group confirmatory factor analysis (MGCFA) to gradually establish measurement invariance levels from configural to strict invariance across all three cohorts.

Because the figural reasoning subtest yields dichotomous data (responses are scored as correct or incorrect), we assessed configural invariance by constraining thresholds and factor loadings of the latent construct to be equal across groups (Wu and Estabrook 2016). Strict invariance was assessed by additionally constraining residual variances to be equal. Model fit was examined based on comparative fit indexes (CFIs). More restrictive models were adopted when between-cohort CFI changes did not exceed 0.01 (Cheung and Rensvold 2002). Subsequently, we estimated latent means and calculated standardized latent change scores across cohorts.

Effect sizes were calculated to indicate the strength of fluid intelligence changes over time, with positive (vs. negative) values representing performance increases (vs. decreases) over the respective interval (i.e., positive vs. negative Flynn effects). Effect sizes were interpreted according to the well-established thresholds introduced by Cohen, being sorted into small, moderate, or large effects (i.e., absolute *d*s = 0.2, 0.5, and 0.8, respectively; Cohen 1988). Cohen *d* values of raw and latent scores were transformed into the IQ metric and IQ changes per decade (DIQ) via the following formula: DIQ (interval) = [(*d* × 15)/interval] × 10 (see Lazaridis et al. 2022). Further, we performed between-cohorts analyses of covariance (ANCOVAs), with respondent sex as a covariate, to assess the potential sex-specificity of the Flynn effect.

All analyses were conducted in R 4.0.2 (R Core Team 2022) and RStudio 2022.07.2+576 (R Studio Team 2022), and measurement invariance analyses were performed with the lavaan R package (Rosseel 2012).

## 3. Results

Our analyses revealed consistent declines in figural reasoning performance over the observed timespan. Measurement invariance analyses showed the good model fit of strict models compared to the configural model (see Table 2), thus suggesting that the BEFKI figural reasoning subscale can be assumed to be fully measurement-invariant across all three (i.e., 2012, 2018, and 2022) cohorts. Therefore, the observed changes can be interpreted as genuine ability changes rather than DIF (e.g., due to changes in test administration format).

**Figure 1:**
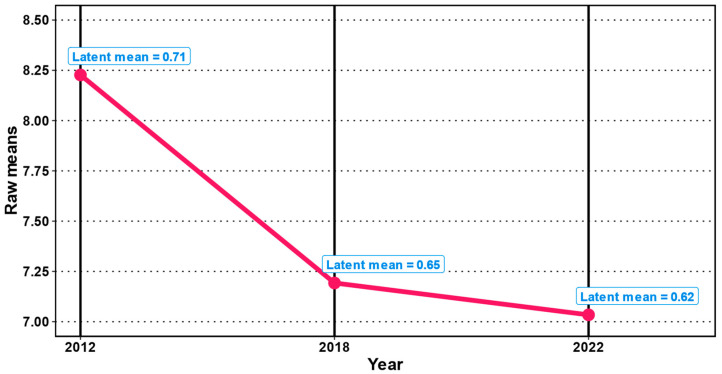
Raw (red) and latent (blue) mean test score changes over the three cohorts.

**Figure 2:**
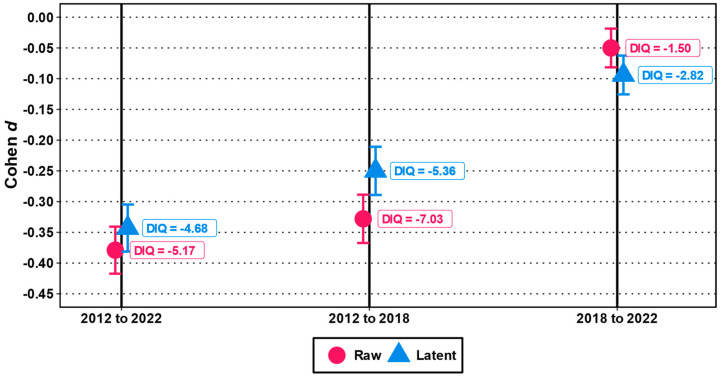
Cohen *d* and DIQ changes between data collection years with 95% confidence intervals.

Standardized test score changes, determined based on raw scores as well as on latent means (see Figure 1), showed consistently significant decreases from 2012 to 2022 (with small-to-medium effect sizes, ranging from *d*s = −0.38 to −0.34; *p*s < 0.001; see Table 3 and Figure 2). These changes amount to a non-trivial loss estimate of 4.68 to 5.17 IQ points per decade.

However, an examination of incremental changes between measurement points showed that the changes appeared to be nonlinear. In the interval between 2012 and 2018, we observed significant decreases in test performance in figural reasoning (*d* = −0.33 and −0.25 for raw scores and latent means, respectively; *p*s < 0.001), representing decreases of about 5.4 to 7.0 IQ points over these six years. Results from the subsequent interval (2018 to 2022) were consistent in terms of effect direction and nominal significance, although only trivial in terms of effect size (*d* = −0.05 and −0.09 for raw scores and latent means, with *p* = < .001 and .01, respectively), corresponding to decreases of 1.5 to 2.8 IQ point over these five years.

Analyses of covariance revealed no statistically significant difference in the observed Flynn effect between boys and girls for any cohort (time by sex *p*s = 0.126 and 0.166 for raw and latent scores, respectively; see Table 4).

## 4. Discussion

Here, we investigated evidence for cross-temporal changes in a measurement-invariant figural reasoning task based on population-representative samples of German secondary school students. Our analyses revealed a reversed (i.e., negative) Flynn effect consistent across all cohorts, although these changes appeared to be nonlinear in terms of effect strength. These findings are interesting because figural reasoning represents a fluid intelligence domain which, on the contrary, typically has been observed to yield the most substantial (positive) Flynn effects over time (for a meta-analysis, see Pietschnig and Voracek 2015).

These findings provide tentative evidence that the recently emerging, rather conflicting, findings about the Flynn effect may be due to the relatively coarse assessments of cognitive performance that have usually been reported in the pertinent literature (see Pietschnig et al. 2023). It could be assumed that more fine-grained assessments (i.e., in terms of CHC-stratum I domains) will beneficially contribute towards clarifying the nature, causes, and meaning of the Flynn effect, as discussed below.

We show non-trivial, measurement-invariant decreases in figural reasoning, which is a central domain of fluid cognitive task performance. This contrasts the global pattern of fluid IQ test scores changes over most of the 1900s (Pietschnig and Voracek 2015). However, recent studies have shown evidence for (partly measurement-invariant) Flynn effect reversals in this very domain in several countries (Austria: Lazaridis et al. 2022; Norway: Bratsberg and Rogeberg 2018; USA: Dworak et al. 2023).

These observations may not solely be attributed to an actual decline in fluid abilities. Instead, studies covering more recent timespans may have investigated test score changes based on more refined intelligence models. They might, therefore, have yielded change scores for more specific cognitive (sub)domains. It thus may be speculated that the past practice of examining IQ test score changes based on distinguishing the rather crude domains of fluid vs. crystallized (and fullscale) IQ sensu Cattell (Cattell 1957) may well have been suboptimal and could inadvertently have masked domain-specific trajectories.

Alternatively, the presently observed unexpected declines may result from a generally reversing Flynn effect globally. In particular, the decreasing strength of the global Flynn effect emerging during the 1980s (Pietschnig and Voracek 2015) has been suggested to be a harbinger of an impending stagnation or even reversal of test score gains. Findings from spatial ability performance changes in Germanophones in recent decades are consistent with this interpretation (Pietschnig and Gittler 2015). However, ambiguous patterns of change within countries and stratum II domains (Lazaridis et al. 2022) suggest a more complex mechanism.

Specifically, it has been argued that changes in ability patterns may result from increased ability differentiation (Pietschnig et al. 2023). According to this idea, one would assume that specific (as opposed to all) abilities are becoming more substantially developed because of the increased specialization of modern-day individuals due to changes in environmental reinforcement. Because *g* is a statistical consequence of the well-established positive manifold of intelligence (Spearman 1904), the ability gain in some specific domains would lead to a weakening of the intercorrelations among IQ subdomains. This, in turn, would explain the previously observed *g*-based decreases (Pietschnig and Voracek 2015; Pietschnig et al. 2023).

However, a decrease in figural reasoning over time cannot be sufficiently explained by ability differentiation because, in its most salient form, ability differentiation would be expected to lead to increases in each subdomain. In contrast, full-scale IQ and the strength of the positive manifold would be expected to decrease. Instead, it may be speculated that ability changes in specific domains may result from changes in environmental demands. Figural reasoning abilities may have become less relevant for success in modern-day environments.

Conceivably, the increasing use of modern technological devices, such as smartphones, tablets, and computers, could have led to individuals (including school students) spending less time on activities that promote figural reasoning (e.g., reading maps, solving puzzles, or drawing; of note, other researchers have argued for the beneficial effects of technology on population IQ developments, see (Neisser 1997)). This would support the gist of previous models postulating IQ changes over time due to social multiplier effects in our ever-changing modern environments (Dickens and Flynn 2001). In this vein, expertise in individual areas is increasingly reinforced through environmental channels, leaving room for genetically based propensities that may promote specialization in a given direction.

Akin to the present results, recent studies have also reported negative Flynn effects in specific domains, such as spatial orientation or working memory capacity (Lazaridis et al. 2022). These findings conform to our observations and may likely be due to a similar mechanism. Modern environments, on the one hand, may reinforce the development of more specific, instead of rather general, ability profiles (but, on the other hand, may no longer reward proficiency in particular specific abilities now seen as obsolete or less expedient). It seems plausible that declines in specific abilities indeed occur. Decreasing task performance in specific domains, such as figural reasoning, could be commensurate with the more general idea of varying and IQ (sub)domain-specific change trajectories, manifesting themselves as differentiated patterns of gain vs. stagnation vs. loss, as evidenced by Lazaridis et al. (2022), that ultimately may lead to a decrease in the strength of the positive manifold of intelligence.

### Strengths and Limitations

The strengths of the current study include the psychometrically unidimensional, measurement-invariant test instrument, the large-scale evidence, the population-representative nature of the samples, and the up-to-datedness of the data. Study limitations to be recognized mainly pertain to several generalizability issues whose relevance is currently unknown: the evidence stems from just one (Western) country, the age range of the test-takers is narrow, the instrument represents a single IQ domain, and amidst the observation period a major technological innovation push—with potential relevance for the topic scrutinized here—took place (in the course of the 2010s, smartphones became ubiquitous).

## 5. Conclusions

In the present study, we show evidence for a negative Flynn effect in figural reasoning on a one-dimensional, measurement-invariant test. These results may indicate that the increasingly inconsistent patterns of the Flynn effect, as witnessed in a growing number of recent reports, may be a consequence of overly broad assessments of cognitive abilities in the datasets typically available for this line of inquiry. It can be speculated that (sub)domain-specific change trajectories are a consequence of changing environmental demands, leading to a decrease in cognitive ability intercorrelations and a weakening of the positive manifold of intelligence.

## Figures and Tables

**Table 1:** Sample characteristics according to cohort.

Data Collected in	2012	2018	2022
*N*	3889	7142	8443
Sex			
Men	1929	3719	4070
Women	1960	3353	4065
Age			
Mean	15.82	15.70	15.60
*SD*	0.29	0.52	0.55

**Table 2:** Model fit across cohorts.

Model	χ^2^	*p*	*df*	CFI
Overall	1337.333	<0.001	104	0.972
2012	334.889	<0.001	104	0.981
2018	492.863	<0.001	104	0.965
2022	664.814	<0.001	104	0.971
Configural	1735.346	<0.001	340	0.968
Strict	1938.521	<0.001	372	0.964

Note. *df* = degrees of freedom; CFI = comparative fit index.

**Table 3:** Raw score- and latent mean-based between-cohort changes, expressed as Cohen *d* and DIQ-values.

Year	2012	2018	2022
2012	-	−0.328 *** (−7.03)	−0.379 *** (−5.17)
2018	−0.250 *** (−5.36)	-	−0.050 ** (−1.50)
2022	−0.343 *** (−4.68)	−0.094 *** (−2.82)	-

Note. The bottom left triangular matrix represents latent mean-based changes, the top right triangular matrix raw score-based changes, and table entries in parentheses are estimated DIQ-values (IQ change per decade). Negative values indicate performance declines over time. ** *p* < .01; *** *p* < .001.

**Table 4:** Model fits of ANOVAs and ANCOVAs based on raw (latent) score calculations.

	*F*	*df*	*p*	η_p_^2^
	ANOVA			
Model fit	*F* = 199.31 (158.52); *df*_1_ = 2, *df*_2_ = 19,471; *p* = < .001			
Time	199.31 (158.52)	2	<0.001 (<0.001)	0.02 (0.02)
	ANCOVA			
Model fit	*F* = 77.90 (61.66); *df*_1_ = 5, *df*_2_ *=* 19,090; *p* = < .001			
Time	192.46 (152.18)	2	<0.001 (<0.001)	0.02 (0.02)
Sex	0.05 (0.31)	1	0.822 (0.512)	<0.001 (<0.001)
Time × Sex	2.07 (0.26)	2	0.126 (0.166)	<0.001 (<0.001)

Note. *df* = degrees of freedom; parenthetical values refer to latent changes.

## Data Availability

The data presented in this study are not publicly available due to privacy restrictions.
